# Ankle syndesmotic ligaments avulsion fractures: incidence in adult population

**DOI:** 10.1186/s13018-024-05156-2

**Published:** 2024-10-12

**Authors:** Xiang Yao, Chong Wang, Weijie Pan, Yicong Chao, Jilei Tang

**Affiliations:** 1 Department of Orthopaedics, The Affiliated People’s Hospital of Jiangsu University, Zhenjiang, Jiangsu 212001 China; 2 Jiangsu University, Zhenjiang, Jiangsu 212001 China; 3 Department of Orthopaedics, Qidong Hospital of Traditional Chinese Medicine, Nantong, Jiangsu 226200 China

**Keywords:** Avulsion fracture, Syndesmotic injuries, Ankle fracture, Anterior inferior tibiofibular ligament, Posterior inferior tibiofibular ligament

## Abstract

**Background:**

Distal tibiofibular syndesmosis injury is often associated with ankle fractures in adults. Injuries to the anterior/posterior inferior tibiofibular ligament (AITFL/PITFL) may present as a mid-substance tear or as an avulsion at insertion. Tibial and fibular avulsion of the AITFL is known as Tillaux fracture and Wagstaffe fracture, respectively. Tibial avulsion of the PITFL is referred to as a Volkmann fracture, and fibular avulsion of the PITFL is still undefined and has not been reported yet. The aim of this study is to summarize the incidence of these four avulsions, that is, tibial and fibular avulsions of the AITFL and PITFL.

**Method:**

Radiography and computed tomography (CT) imaging data of all adult patients with ankle fractures treated at our hospital between November 2010 and March 2023 were retrospectively analyzed. All ankle fractures were classified according to the Weber-AO and Lauge-Hansen classification systems by two experienced radiologists and two surgeons. The incidence of the four avulsions of the AITFL/PITFL was determined.

**Results:**

In total, 1,770 ankle fractures in 1,758 patients were included in this study. The total incidence of avulsions at the four insertions of the AITFL/PITFL (occurring at one, two, or three insertions) was found to be 26.3% (465/1,770). Volkmann fracture had the highest incidence (19.9%, 353/1,770), and it was followed by Tillaux fracture (5.3%, 93/1,770), Wagstaffe fracture (3.3%, 59/1,770), and fibular avulsion of the PITFL (0.5%, 8/1,770). It is noteworthy that fibular avulsion of the PITFL has been reported here for the first time. The incidence of avulsion at one insertion was 23.6% (418/1770) and 2.7% (47/1770) at multiple insertions.

**Conclusion:**

In adult ankle fractures, avulsion occurs at the four insertions of the AITFL/PITFL in more than 25% of patients. The tibial insertion of the PITFL had the highest incidence of avulsion among the four insertions, while the fibular insertion of the PITFL had the lowest. The four types of avulsions can be isolated or in association with other avulsions. Future research studies on these four types of avulsion fractures would help in accurate diagnosis, decision-making and treatment of ankle Syndesmosis injuries.

**Level of evidence:**

Level III, retrospective cohort study.

## Introduction

Ankle fractures are one of the most common types of lower extremity fractures that account for approximately 10% of all fractures [1]. Ankle fractures are commonly associated with injuries to the distal tibiofibular syndesmosis, which is a significant structure in maintaining the stability of the ankle joint. The distal tibiofibular syndesmosis consists of the distal tibia, distal fibula, and four ligaments, namely, the anterior inferior tibiofibular ligament (AITFL), the posterior inferior tibiofibular ligament (PITFL), the interosseous ligament, and the interosseous membrane. Each anatomical structure plays a synergistic role in maintaining the stability of the tibiofibular syndesmosis, and their contribution to its stability is as follows: interosseous ligament, 22%; AITFL, 35%; superficial PITFL, 9%; and deep PITFL, 33% [2]. Among these, the AITFL primarily restrains external rotation of the fibula, while the PITFL serves as the principal restraint against posterior translation [3].

**Fig. 1:**
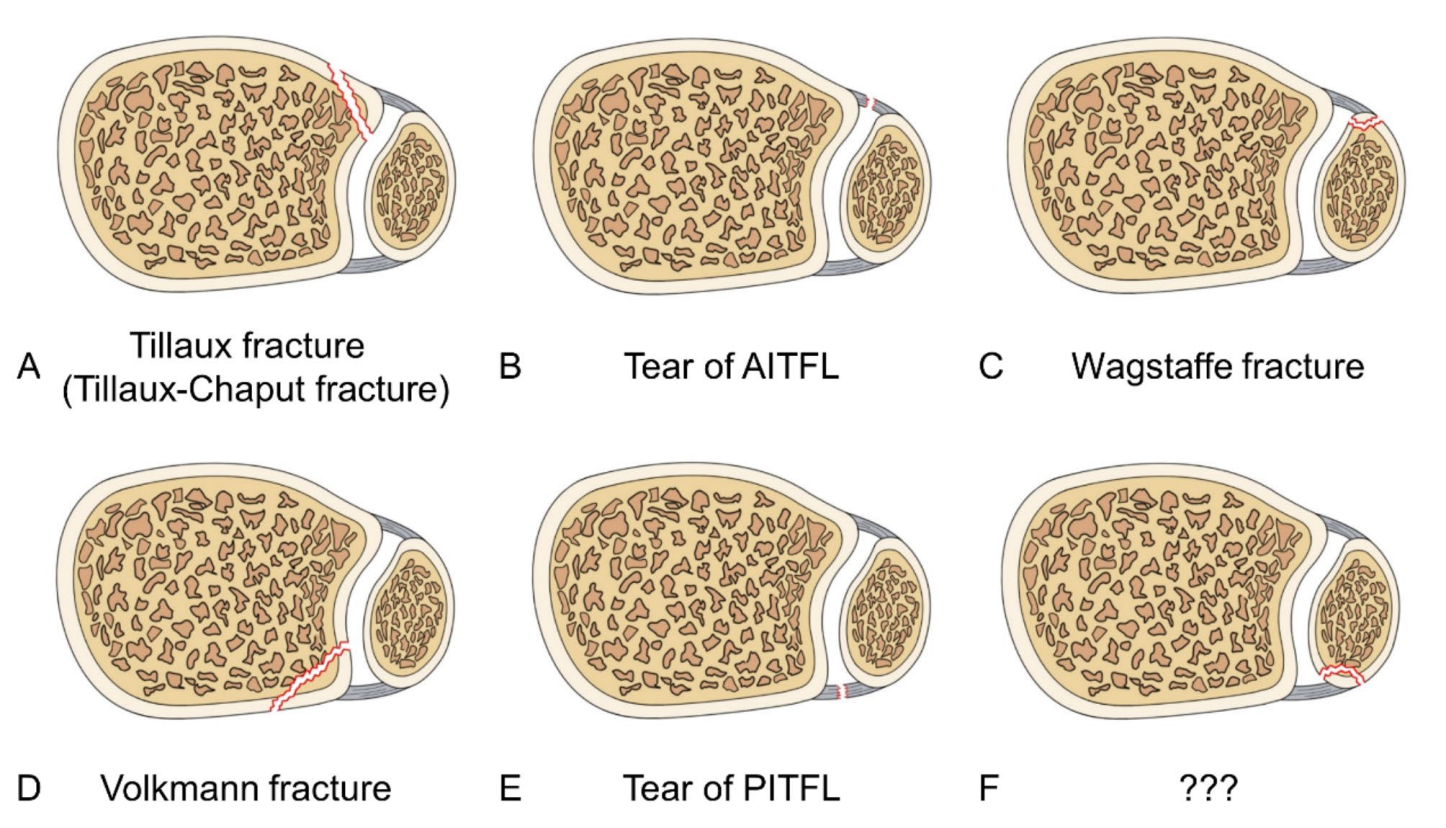
Schematic diagram showing the six types of the anterior/posterior inferior tibiofibular ligament (AITFL/PITFL) injuries that can occur individually or simultaneously. **A**. Tibial avulsion of the AITFL (Tillaux fracture or Tillaux-Chaput fracture). **B**. Substance tear of the AITFL. **C**. Fibular avulsion of the AITFL (Wagstaffe fracture). **D**. Tibial avulsion of the PITFL (Volkmann fracture). **E**. Substance tear of the PITFL. **F**. Fibular avulsion of the PITFL (undefined). AITFL/PITFL: anterior/posterior inferior tibiofibular ligament

AITFL/PITFL may be subjected to different levels of stretch that eventually result in mid-substance tear (Fig. 1B & E) or avulsion at its four insertions, that is, the tibial and fibular avulsions of the AITFL and PITFL respectively. Tibial avulsion of the AITFL was first reported by Cooper [4] and is now often referred to as Tillaux fracture or Tillaux-Chaput fracture [5] (Fig. 1A). Fibular avulsion fracture of the AITFL is called Wagstaffe fracture [6] (Fig. 1C). Tibial avulsion of the PITFL was first reported by Earle [7] and is now commonly referred to as Volkmann fracture [8] (Fig. 1D). However, fibular avulsion of the PITFL has not been reported so far (Fig. 1F). These avulsion fragments are characterized by large morphological variations and cannot easily be detected on plain radiographic film. Moreover, the commonly used Weber-AO classification and Lauge-Hansen classification for ankle fractures fail to include information on these four avulsion injuries [9, 10]. Failure to repair the single or multiple major avulsions is likely to result in chronic ankle instability of the tibiofibular syndesmosis and post-traumatic osteoarthritis [11]. Thus, accurate identification and proper management of these four specific avulsions are helpful for restoring the stability of the distal tibiofibular syndesmosis, ensuring that it does not progress to a chronic condition, and improving the prognosis of ankle fractures. The incidence data of avulsions is helpful for clinicians to pay attention to and identify special avulsion fractures, avoid missing important bone fragments. However, there is no comprehensive summary in the literature on avulsions of the AITFL/PITFL in the adult population. Therefore, the purpose of this study was to summarize the incidence and characteristics of the above-mentioned four avulsions of the AITFL/PITFL in adult patients with ankle fractures. This is conducive to preventing missed diagnoses, helping clinicians formulate better treatment plans, and improving the cure rate of ankle avulsion fractures.

## Methods

This study gathered and analyzed radiography and three-dimensional (3D) computed tomography (CT) images of all adult patients with closed ankle fractures admitted to the Affiliated People’s Hospital of Jiangsu University between November 2010 and March 2023. The exclusion criteria were as follows: age less than 18 years, open injury, congenital malformations, pathological fractures, explosive wound, gunshot wound, and insufficient radiographic data. A total of 1,758 patients (986 males and 772 females) were included. This study was approved by the Ethics Committee of the Affiliated People’s Hospital of Jiangsu University. The imaging data were reviewed by four reviewers, including two orthopedic trauma surgeons and two radiologists. None of the reviewers had conflicts of interest with the patients. All ankle fractures were classified using the Weber-AO and Lauge-Hansen classification system.

The two-dimensional and three-dimensional CT images of all patients were independently reviewed by four researchers. Avulsion was diagnosed in cases where a typical fractures or free fragment was observed at the point of attachment of the tibia and fibula of the AITFL/PITFL. Four specific avulsions (Tillaux fracture, Wagstaffe fracture, Volkmann fracture, and fibular avulsion of the PITFL) were identified and diagnosed unanimously by three observers. If their diagnosis was inconsistent in any case, they discussed it several times until the majority agreed on a diagnosis.

Statistical analysis was performed using the IBM SPSS Statistics 26 software (IBM Corporation, Armonk, NY, USA). Qualitative data are expressed as numerical values and percentages, and quantitative data are expressed as mean ± SD values.

## Results

In total, 1,758 patients with 1,770 ankle fractures were retrospectively analyzed in this study. Among them, 986 (56.1%) were males and 772 (43.9%) were females. The average age was 51.1 ± 15.0 years (males: 48.9 ± 14.8 years, females: 53.8 ± 14.8 years). There were 834 left ankle injuries, 912 right ankle injuries, and 12 cases of bilateral ankle injuries. The most common cause of injury was ankle sprain (40.5%, 716/1770), followed by traffic accidents and collision injuries (31.2%, 552/1770), fall injuries (24.5%, 433/1770), and other injuries (3.9%, 69/1770). According to the Weber-AO classification, there were 553 type A cases (31.2%), 654 type B cases (36.9%), 276 type C cases (15.6%), and 287 cases (16.2%) that could not be classified due to absence of fibular fractures. According to the Lauge-Hansen classification, there were 189 cases of pronation-abduction injury (10.7%), 242 cases of pronation-external rotation injury (13.7%), 663 cases of supination-external rotation injury (37.4%), 545 cases of supination-adduction injury (30.8%), and 131 (7.4%) cases of simple posterior ankle fractures or pilon fractures, which could not be properly classified by the Lauge-Hansen classification (Table 1).

**Table 1:** Demographic characteristics of patients and fractures

	Characteristics	
Patients (*n* = 1758)	Sex ratio (Male/Female)	986/772(1:0.78)
	Age(±SD, years)	51.06 ± 14.99
	Age(Male/Female)	48.93 ± 14.82/53.80 ± 14.78
Ankles (*n* = 1770)	Left only	834(47.1%)
	Right only	912(51.5%)
	Bilateral	12(0.7%)
	Left/Right	846/924(1:1.09)
Weber-AO classification	A	553(31.2%)
	B	654(36.9%)
	C	276(15.6%)
	absence of fibula fractures	287(16.2%)
Lauge-Hansen classification	supination-adduction	545(30.8%)
	supination-external	663(37.4%)
	pronation-abduction	189(10.7%)
	pronation-external	242(13.7%)
	could not be classified	131(7.4%)

**Fig. 2:**
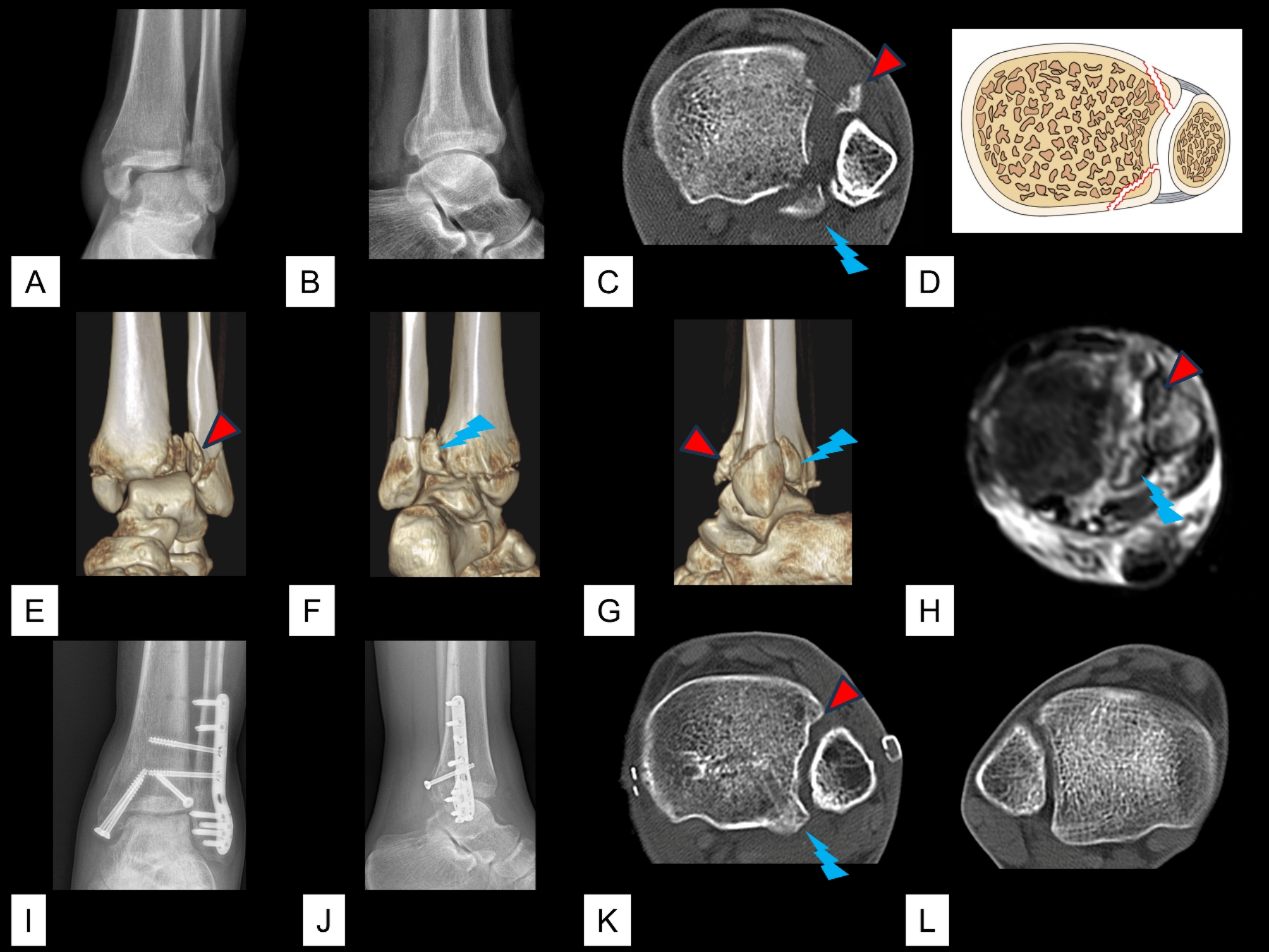
An ankle fracture concomitant with Tillaux fracture and Volkmann fracture. **A** & **B**. Preoperative radiographic images. **C**. Preoperative axial CT image. **D**. Schematic diagram of this type of injury. **E**–**G**. Preoperative 3D CT images. **H**. Preoperative magnetic resonance imaging images. **I**–**J**. Postoperative radiographic images. **K** & **L**. Axial CT images after removal of the implant. Red triangle: Tillaux-Chaput fracture; blue arrow: Volkmann fracture

**Fig. 3:**
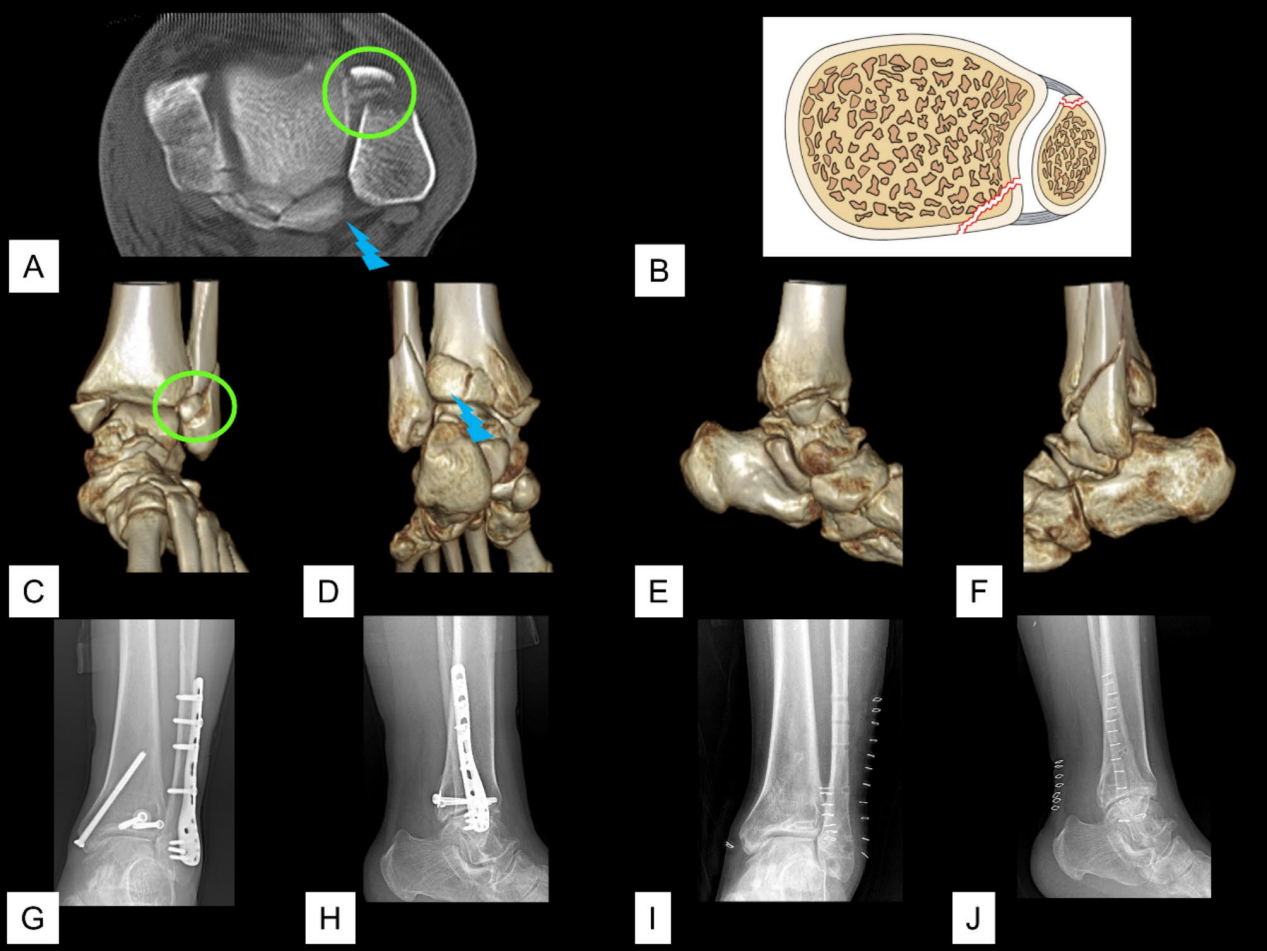
An ankle fracture concomitant with Wagstaffe fracture and Volkmann fracture. **A**. Preoperative axial CT image. **B**. Schematic diagram of this type of injury. **C**–**F**. Preoperative 3D CT images. **G** & **H**. Postoperative radiographic images. **I** & **J**. Radiographic images after removal of the implant. Green circle: Wagstaffe fracture; blue polyline: Volkmann fracture

**Fig. 4:**
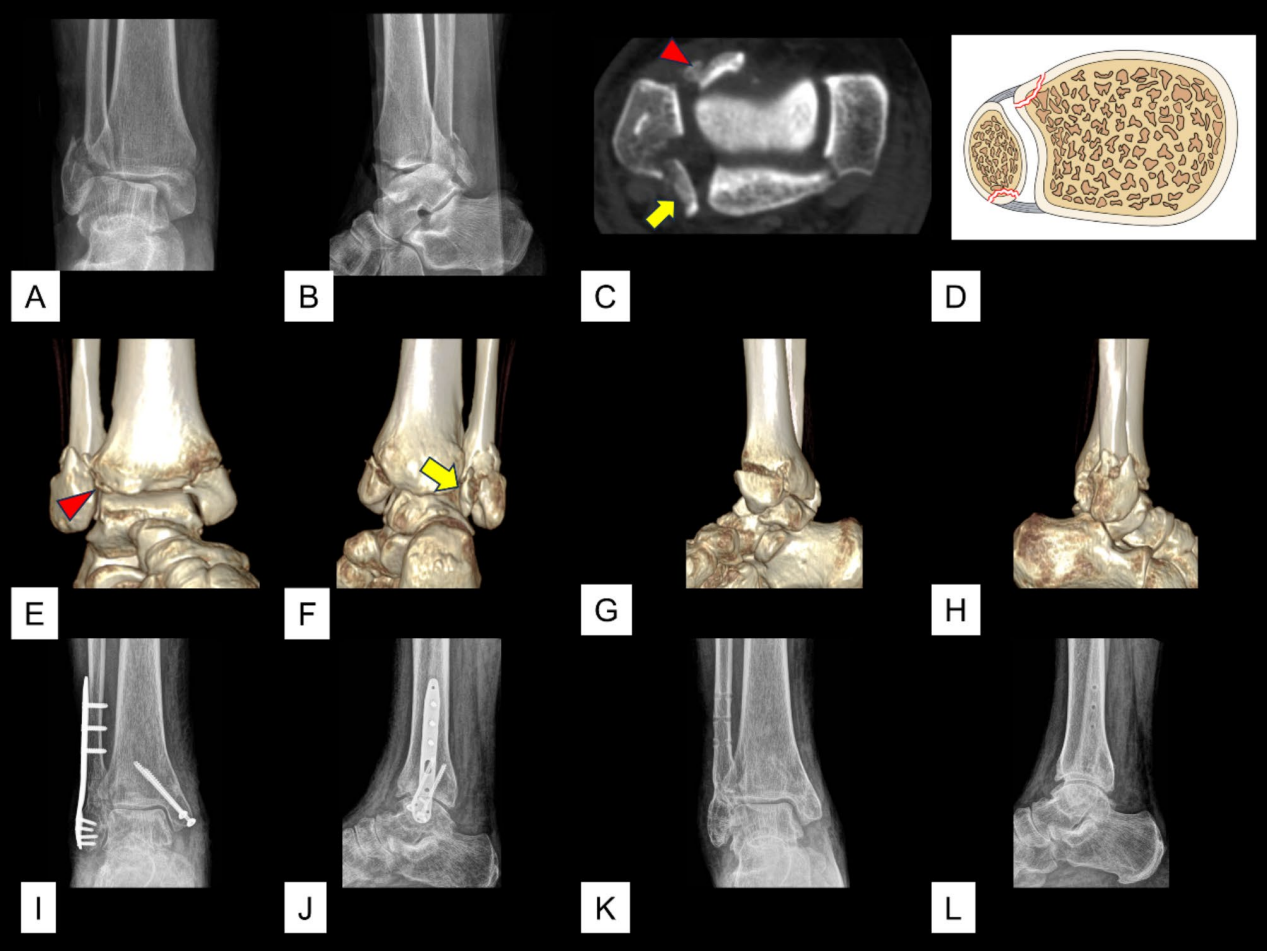
A case of fibular avulsion of the PITFL associated with a Tillaux-Chaput fracture and a lateral malleolar fracture. A & B. Preoperative radiographic images. C. Preoperative axial CT image. D. Schematic diagram of this type of injury. E–H. Preoperative 3D CT images. I & J. Preoperative radiographic images. K & L. Radiographic images after removal of the implant. Red triangle: a Tillaux fracture; yellow arrow: a fibular avulsion of the PITFL

**Fig. 5:**
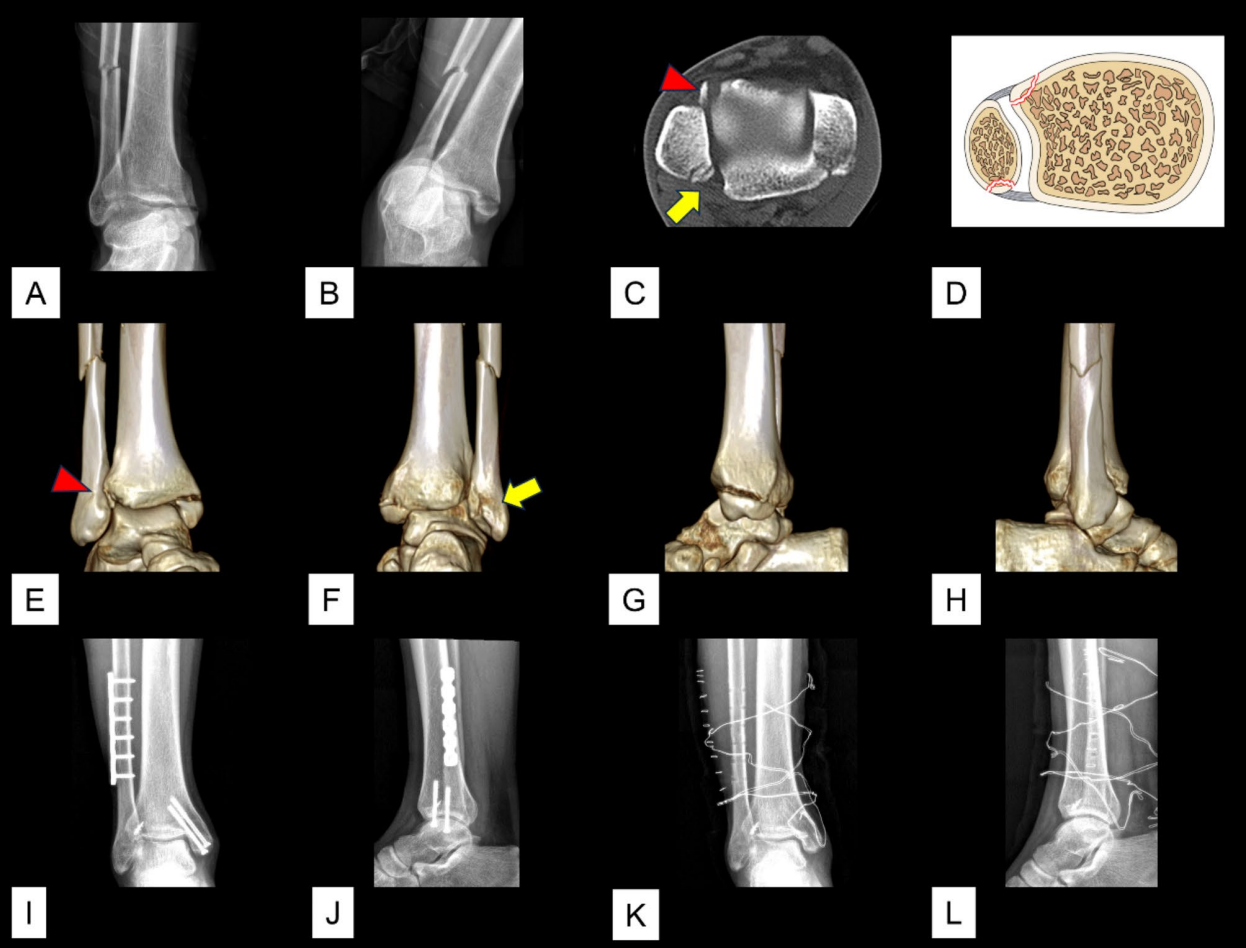
A case of fibular avulsion of the PITFL associated with a Tillaux-Chaput fracture and fibular shaft fracture. **A** & **B**. Preoperative radiographic images. **C**. Preoperative axial CT image. **D**. Schematic diagram of this type of injury. **E**–**H**. Preoperative 3D CT images. **I** & **J**. Preoperative radiographic images. **K** & **L**. Radiographic images after removal of the implant. Red triangle: a Tillaux fracture; yellow arrow: a fibular avulsion of the PITFL

The total incidence of avulsion of the AITFL/PITFL at the four insertions was 26.3% (465/1770). Of the 465 cases of avulsion, the avulsion was observed at one insertion in 418 cases, at two insertions in 46 cases, and at three insertions in 1 case. The 46 (2.6%, 46/1,770) cases of avulsions that occurred at two insertions included 23 (1.3%, 23/1,770) cases of Tillaux fracture concomitant with Volkmann fracture (Figs. 2), 20 (1.1%, 20/1,770) cases of Wagstaffe fracture concomitant with Volkmann fracture (Fig. 3), and 3 (0.2%, 3/1,770) cases of Tillaux fracture concomitant with fibular avulsion of the PITFL (Figs. 4 and 5). Volkmann fracture had the highest incidence (19.9%, 353/1,770), followed by Tillaux fracture (5.3%, 93/1,770), Wagstaffe fracture (3.3%, 59/1,770), and fibular avulsion of the PITFL (0.5%, 8/1,770).

**Fig. 6:**
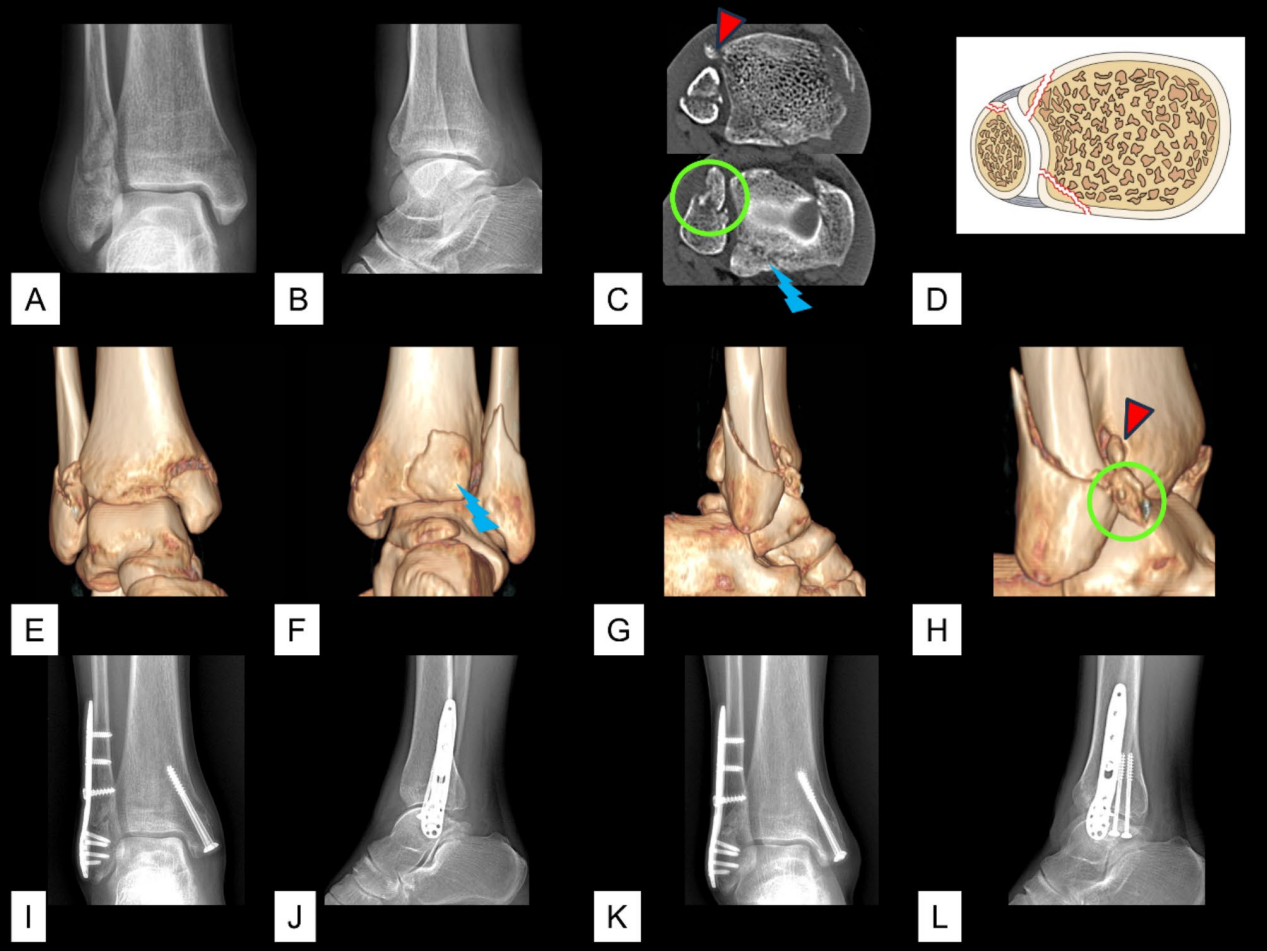
A rare ankle fracture with avulsions at three insertions, that is, a Tillaux fracture, a Wagstaffe fracture, and a Volkmann fracture combined **A** & **B**. Preoperative radiographic images. **C**. Preoperative axial CT image. **D**. Schematic diagram of this type of injury. **E**–**H**. Preoperative 3D CT images. **I**–**L**. Postoperative radiographic images. Red triangle: a Tillaux-Chaput fracture; green circle: a Wagstaffe fracture; blue arrow: a Volkmann fracture

Finally, we found one case of avulsion that occurred at three insertions (Fig. 6): a Tillaux fracture combined with a Wagstaffe fracture and a Volkmann fracture. This is a rare injury with an incidence of 0.1% (1/1,770).

## Discussion

In this study, we analyzed the radiography and CT images of a large sample of patients with ankle fractures and summarized the characteristics of four specific avulsions of the AITFL/PITFL. We detected avulsions of the AITFL/PITFL in 465 out of 1,770 ankle fractures, with a total incidence of 26.3%. Avulsions at the four insertions were most commonly found in supination-external rotation fractures, which accounted for approximately two-thirds (39/93, 39.8%) of Tillaux fractures and fibular avulsions of the PITFL, three-quarters (43/59, 72.9%) of Wagstaffe fractures, and two-thirds (231/353, 65.4%) of Volkmann fractures (Table 2). Avulsion of the AITFL/PITFL may occur at one insertion or at multiple insertions. The incidence of avulsion at one insertion was 23.6% (418/1770) and 2.7% (47/1770) at multiple insertions. In this study, avulsions were found at one, two, or three insertions, but there were no cases of avulsion at all four insertions. Most of the avulsions (418/1,770) occurred at one insertion, and the Volkmann fracture had the highest incidence of all four avulsions (19.9%, 353/1,770). This could be attributable to the synergistic effect of the traction of the PITFL and the posterior superior impact of the talus. The incidence of avulsion at two insertions was 2.6% (46/1770), including 23 cases of Tillaux fracture combined with Volkmann fracture (Figs. 2), 20 cases of Wagstaffe fracture combined with Volkmann fracture (Fig. 3), and three cases of Tillaux fracture combined with fibular avulsion of the PITFL (Figs. 4 and 5). Kose et al. reported two cases of isolated adult Tillaux fracture associated with Volkmann fracture and suggested that such fractures are caused by strong external rotation force of the foot [12]. In line with their speculation, in this study, 16 of the cases of Tillaux fracture combined with Volkmann fracture occurred under external rotation force. Moreover, a similar case of Tillaux fracture occurring in combination with Volkmann fracture as a result of external rotation force was also reported by Mansur et al. [13].

**Table 2:** Demographic characteristics of patients with avulsions

		Tillaux fracture	Wagstaffe fracture	Volkmann fracture	Fibular avulsion of the PITFL	Total
Sex	Male	48	29	179	5	261
	Female	45	30	174	3	252
Side	Left	45	28	170	4	247
	Right	48	31	183	4	266
Lauge-Hansen classification	Supination-adduction	12 (2.3%)	8 (1.6%)	13 (2.5%)	3 (0.6%)	36 (7.0%)
	Supination-external	37 (7.2%)	43 (8.4%)	231 (45.0%)	3 (0.6%)	314 (61.2%)
	Pronation-abduction	11 (2.1%)	2 (0.4%)	63 (12.3%)	0 (0%)	76 (14.8%)
	Pronation-external	25 (4.9%)	5 (1.0%)	25 (4.9%)	2 (0.4%)	57 (11.1%)
	Could not be classified	8 (1.6%)	1 (0.2%)	21 (4.1%)	0 (0%)	30 (5.8%)
	Total	93 (18.1%)	59 (11.5%)	353 (68.8%)	8 (1.6%)	513
Weber-AO classification	A	12 (2.3%)	8 (1.6%)	14 (2.7%)	3 (0.6%)	37 (7.2%)
	B	37 (7.2%)	43 (8.4%)	230 (44.8%)	3 (0.6%)	313 (61.0%)
	C	31 (6.0%)	7 (1.4%)	69 (13.5%)	2 (0.4%)	109 (21.2%)
	Could not be classified	13 (2.5%)	1 (0.2%)	40 (7.8%)	0 (0%)	54 (10.5%)
	Total	93 (18.1%)	59 (11.5%)	353 (68.8%)	8 (1.6%)	513

The Tillaux fracture appears on CT images as a small fragment of the anterior tibiofibular ligament breaking off from its attachment on the anterolateral tibia. Stefan et al.. proposed that Tillaux-Chaput fractures can be divided into three types: (1) extra-articular avulsion of the AITFL; (2) fracture of the anterolateral tibia involving the joint and tibial incisura; and (3) fracture with impaction of the anterolateral tibial plafond [14]. Tillaux fractures are most common in adolescents between 12 and 14 years of age [15], and it is therefore also referred to as juvenile Tillaux fracture. It represents a transitional subset of Salter-Harris type III fractures of the anterolateral tibial epiphysis [16]. Previous studies have shown that the incidence of Tillaux fractures in adolescents is 5.2–10.0% [17]. However, the incidence of isolated Tillaux fractures in adults has not been reported to date. There have only been a few case reports of adult Tillaux fractures in the literature. Syed et al. [18]., Mishra et al. [19]., and Millan et al. [20]. reported 1 case each of adult Tillaux fracture, and Pereira et al.. reported 2 cases of isolated adult Tillaux fracture [21]. In our study, we identified 93 patients with concomitant Tillaux fractures among the 1,770 ankle fractures, with an incidence of 5.3%. Tillaux fractures were found to occur in conjunction with Volkmann fractures (20 cases) and fibular avulsions of PITFL (3 cases), but there were no cases of combined Wagstaffe fractures. Based on our data, the incidence of Tillaux fractures in adults appears to be significantly lower than that in adolescents. This discrepancy can be attributed to adults having reached full skeletal maturity, as a result of which there is no vulnerable area in the point at which the epiphysis connects with the bone shaft. Thus, when the adult ankle joint is subjected to external forces, the AITFL is more likely to tear than to avulse at its insertion [22]. Tillaux fractures are not easily detected on radiographic images, and it has been suggested that oblique ankle views should be obtained in patients with suspected fracture, along with stress radiography and magnetic resonance imaging to aid in the diagnosis if necessary [23]. Shrestha reported a case of a Tillaux-Chaput fracture diagnosed by ultrasonography and pointed out that the key to diagnosing a fracture by ultrasonography lies in recognizing the cortical discontinuity along the surface of the bone [24]. The treatment of Tillaux fractures is dependent on the size of the bone mass and the degree of displacement. Tillaux fractures with less than 2 mm of displacement can be treated in a conservative way. However, when fragment displacement is > 2 mm or translation is > 1 mm, the patient requires open reduction and internal fixation, including arthroscopic-assisted treatment [25], screw fixation [26], and tension band fixation [27]. A recent study indicates that suture button fixation may yield better outcomes compared to syndesmotic screw fixation [28].

On CT, the Wagstaffe fracture presents as an avulsion fragment of the anteromedial aspect of the distal fibula, which must be distinguished from an avulsion of the lateral collateral ligament. To identify a Wagstaffe fracture, it needs to be considered in conjunction with the patient’s medical history and physical examination, and the use of 3D CT can lead to more accurate identification of such injuries. Wagstaffe classified the Wagstaffe fracture into three types: type I, displaced avulsion fracture of the distal end of the fibula; type II, fracture of the anterior spike of the proximal fibular fragment; type III, fractures of the anterior tubercles from both the tibia and fibula [6]. Wagstaffe fracture often occurs concomitantly with other ankle injuries, such as medial malleolus fractures and fibula fractures [29], with its incidence ranging from 2.7% [29] to 26.5% [30]. Park et al.. reported 13 cases of Wagstaffe fracture, with an incidence of 25% (13/52) [31], and Fisher et al.. reported 40/151 (26.5%) cases [30]. In this study, a total of 59 Wagstaffe fractures were found of which 1 (0.1%, 1/1,770) was classified as type I and 58 (3.3%/1,770) were classified as type II. Wagstaffe fractures were found to occur in conjunction with Volkmann fractures (20 cases), while there were no cases of Tillaux fractures combined with fibular avulsions of the PITFL. The incidence of Wagstaffe fractures in our study was 3.3% (59/1,770), which is lower than that reported in previous studies. This is probably attributable to the large sample size used in this study and the fact that we analyzed all types of ankle fractures rather than only a single type. With regard to the management of Wagstaffe fractures, the size of the fragment influences the fixation method. When the fragment is 5 mm or larger, it needs to be fixed directly, and a non-absorbable suture, Kirschner wire, or single screw can be used for fixation [32].

Volkmann fracture presents as a posterior-lateral avulsion fragment of the distal tibia that is primarily caused by excessive pulling of the PITFL and posterior and superior impact on the talus. The shape and size of a posterior malleolus fracture can often only be determined by spiral CT 3D reconstruction technology. The Volkmann fracture is a type of posterior malleolus fracture that can be classified under Haraguchi type I fractures and accounts for 67% of all posterior malleolus fractures [8]. Volkmann fractures have been rarely reported to occur in isolation in previous studies. Heim et al.. reported 36 cases of Volkmann fracture and discussed its treatment methods [33]. In this study, Volkmann fractures were observed in 353 out of 1,770 adult ankles, with an incidence of 19.9%, which was the highest among the four insertions around the tibiofibular syndesmosis. Volkmann fractures occurred in conjunction with Tillaux fractures (23 cases) and Wagstaffe fractures (20 cases), but no cases of Volkmann fractures combined fibular avulsions of the PITFL were found. The treatment of Volkmann fractures is dependent on the size of the fragment and the degree of displacement. When the posterior fragment is < 10% of the distal articular surface, the treatment is mainly conservative. However, when the posterior fragment is > 25% of the articular surface or displaced by > 2 mm, it is treated with open reduction and internal fixation [34]. It has been suggested that a fragment in the size range of 10–25% does not require fixation of the posterior ankle if the articular surface is flat. However, posterior fragment fixation was reported to contribute to functional outcomes by decreasing the incidence of articular step-off of > 1 mm [35]. Therefore, it might be beneficial in the management of Volkmann fractures. Fixation of Volkmann fracture can be achieved with screws [36] and plates [8]. Ahmed et al. advocated that in cases of posterior malleolus fractures accompanied by syndesmotic diastasis, the posterior malleolus should be fixed first, as this approach may eliminate the need for syndesmotic screws [37]. Giordano et al.. suggested that for fragments with 10% involvement of the tibiotalar articular surface, the use of a trans-syndesmotic screw is sufficient, but when the involvement is greater than 25%, either a non-locked or a locked plate must be used to buttress the tibial posterior malleolus. In posterior fragments with 45% involvement, the use of a locking plate is recommended [34].

A remarkable finding in this study is that there were 8 (0.5%) cases of fibular avulsion of the PITFL among the 1,770 adult ankle fractures. To our knowledge, this is the first report of this type of fracture in the current literature. This avulsion appeared as a posterior medial avulsion fragment of the distal fibula on CT images, but lacked specificity on radiographic images. In our study, all eight cases of fibular avulsion fractures of PITFL were diagnosed by CT imaging (including two-dimensional and three-dimensional). Among the 8 patients, there were 5 males and 3 females, with an average age of 58.5 ± 11.0 years. According to the Weber-AO classification system, there were three cases of type A, three cases of type B, and two cases of type C fractures. According to the Lauge-Hansen classification system, there were three cases of supination-adduction type fractures, three cases of supination external rotation type fractures, and two cases of pronation external rotation type fractures (Table 3). Three of the eight cases were associated with Tillaux-Chaput fractures and five were isolated. Six of the eight patients were treated surgically, and two were treated conservatively with cast immobilization. Six of the eight patients underwent surgery, but none underwent targeted fixation of the bone fragment. Further, three of the eight fibular avulsions of the PITFL were accompanied with Tillaux fractures (Figs. 4 and 5).

**Table 3:** Demographic characteristics of patients with fibular avulsion of the PITFL

	Characteristics	
Patients (*n* = 8)	Sex ratio (Male/Female)	5:3
	Age (mean ± SD), years	58.5 ± 11.0
	Age (Male/Female)	53.4 ± 8.0/67.0 ± 11.3
Ankles (*n* = 8)	Left	4 (50.0%)
	Right	4 (50.0%)
	Left/Right	4/4 (1:1)
Weber-AO classification	A	3 (37.5%)
	B	3 (37.5%)
	C	2 (25.0%)
	Absence of fibula fractures	0 (0%)
Lauge-Hansen classification	Supination-adduction	3 (37.5%)
	Supination-external	3 (37.5%)
	Pronation-abduction	0 (0%)
	Pronation-external	2 (25.0%)
	Could not be classified	0 (0%)

Fibular avulsion of the PITFL is theoretically similar to the Volkmann fracture, which is also caused by separation of the tibiofibular syndesmosis. According to Neer’s ring theory [38], a fibular avulsion of the PITFL also implies disruption of the integrity of the ankle joint ring. When missed or left untreated, this avulsion fracture may lead to ankle instability, pain, and other complications, which may affect the restoration of normal ankle joint anatomy and function. Appropriate management of this newly identified fracture type will certainly contribute to the stability of the lower tibiofibular syndesmosis of the ankle. Neither the Weber-AO or Lauge-Hansen classification describe the morphology or injury mechanism of this type of fracture. Thus, a more comprehensive classification system of ankle fractures that includes this avulsion fracture is required. Regrettably, due to its extremely low incidence, the treatment strategy for this type of fracture is unclear and warrants further research.

## Limitations

There are some limitations to this study that need to be mentioned. First, the sample size is limited, and a larger sample would help determine more accurate incidence rates from which more sound conclusions can be drawn. Second, this study only analyzed injuries to bony structures and did not focus on injuries related to substance tears of ligaments. MRI data could provide more information about soft tissue injuries. Third, the relationship between the number/position of the four avulsions and the stability of the distal tibiofibular syndesmosis warrants further investigation. Fourth, the fixation methods of the newly discovered fibular avulsion of the PITFL need to be validated through appropriate biomechanical testing.

## Conclusion

Based on the present findings, we can conclude that in adult ankle fractures, more than 25% of patients are likely to experience avulsion at the four insertions of the AITFL/PITFL. In this sample, the tibial insertion of the PITFL had the highest incidence (19.9%, 353/1,770) of avulsion among the four insertions, while the fibular insertion of the PITFL had the lowest (0.5%, 8/1,770). Further study of these four avulsions would be beneficial in terms of precise diagnosis and treatment decision-making in ankle fracture injuries.

## Data Availability

No datasets were generated or analysed during the current study.
